# Effect of Thrombopoietin Receptor Agonist on Pregnant Mice

**DOI:** 10.3390/pharmaceutics14030514

**Published:** 2022-02-25

**Authors:** Kensaku Nakai, Takuya Misugi, Kohei Kitada, Yasushi Kurihara, Mie Tahara, Akihiro Hamuro, Akemi Nakano, Masayasu Koyama, Yukimi Kira, Daisuke Tachibana

**Affiliations:** 1 Department of Obstetrics and Gynecology, Osaka City University Graduate School of Medicine, 1-4-3 Asahimachi Abeno-ku Osaka, Osaka 545-8585, Japan; m1388253@med.osaka-cu.ac.jp (T.M.); k-kitada@med.osakacity-hp.or.jp (K.K.); y.kuri@med.osaka-cu.ac.jp (Y.K.); mtahara@med.osaka-cu.ac.jp (M.T.); m1156991@med.osaka-cu.ac.jp (A.H.); akeake@med.osaka-cu.ac.jp (A.N.); m-koyama@med.osaka-cu.ac.jp (M.K.); dtachibana@med.osaka-cu.ac.jp (D.T.); 2 Department of Research Support Platform, Osaka City University Graduate School of Medicine, 1-4-3 Asahimachi Abeno-ku Osaka, Osaka 545-8585, Japan; yukimi@med.osaka-cu.ac.jp

**Keywords:** immune thrombocytopenia (ITP), pregnancy, thrombopoietin receptor agonists (TPO-RAs), romiplostim

## Abstract

Thrombopoietin receptor agonists (TPO-RAs) are an effective treatment for refractory immune thrombocytopenia (ITP). However, the use of TPO-RAs is limited for ITP in pregnant women due to concerns about fetal toxicity. In this study, we examined the effects of romiplostim, one of the TPO-RAs, on pregnant mice. The mice were injected subcutaneously with romiplostim (1, 5, 10, 30, and 100 μg/kg) on gestational days (GD) 1, 8, and 15. We evaluated maternal and fetal platelet and megakaryocyte counts (MK), fetal weight at birth, placental morphology, and miscarriage rates. Romiplostim increased platelet and MK counts in pregnant mice at all doses and in fetuses at doses above 10 µg/kg. Fetal weight at birth was slightly reduced at a dose of 100 μg/kg, but there were no significant differences in placental weight, spiral artery wall thickness, placental growth factor signal changes, or the rate of resorption at that dosage. The dose of romiplostim used clinically for ITP patients (1–10 μg/kg) did not show any adverse effects on pregnant mice. Although the results of the present study are encouraging, until there are more conclusive data, the use of romiplostim should be evaluated individually in severe, life-threatening cases, and all relevant ethical aspects should be considered.

## 1. Introduction

Immune thrombocytopenia (ITP) is an autoimmune disease characterized by persistent thrombocytopenia due to the binding of antibodies to platelet antigens and the premature destruction of platelets by the reticuloendothelial system, mainly in the spleen [1]. ITP often occurs in women between the ages of 20 and 40 years and is therefore often associated with pregnancy or diagnosed only after pregnancy. ITP occurs in 1–10 women per 10,000 pregnancies and accounts for 5% of all cases of pregnancy-related thrombocytopenia [2,3]. ITP is often exacerbated during pregnancy [4], and it is important to maintain a safe maternal platelet count because purpura, epistaxis, and gastrointestinal bleeding may occur with a severe decrease in platelet count, thus increasing the risk of postpartum hemorrhage [5].

The first-line therapy for pregnant women with ITP recommended by the American Society of Hematology guidelines is either intravenous immunoglobulin (IVIg) or corticosteroids [6], but there are scattered cases of refractory ITP that do not respond to treatment. Azathioprine, cyclosporine, and rituximab are used for the treatment of refractory ITP, but the use of these drugs is limited in pregnant women because of their impact on the fetus [7]. In 2008, thrombopoietin receptor agonists (TPO-RAs) were approved in the U.S., and they have significantly improved the treatment of ITP in adults and are now a second-line therapy [6]. TPO-RAs bind to the TPO receptor (c-Mpl), which is expressed on megakaryocytes and hematopoietic stem cells and enhances platelet production by promoting megakaryocyte differentiation and maturation [8]. However, despite relatively established strategies for treating serious disease and the advent of this therapeutic option, the use of TPO-RAs during pregnancy is still limited [9]. Furthermore, the U.S. Food and Drug Administration (FDA) has placed TPO-RAs in category C for use during pregnancy, meaning that animal studies have shown adverse effects on the fetus. However, there have been no well-controlled studies in human pregnant women thus far [10].

Although an increase in platelets has been observed in a study in which TPO-RAs were administered to non-pregnant mice [11], a basic study of TPO-RAs administered to pregnant mice has not yet been reported. In the present study, we administered romiplostim, one of the four TPO-RAs, to pregnant mice and examined its effects on maternal and fetal platelet counts, morphological changes in maternal femoral bone marrow, fetal liver and placenta, fetal weight at birth, and miscarriage rate.

## 2. Materials and Methods

### 2.1. Mice

All protocols and experimental procedures were performed in accordance with the guidelines of the Institutional Review Board and approved by the ethical committee of the Institutional Animal Care and Use Committee of Osaka City University on 24 December 2020 (approval number: 20028). Pregnant ICR mice (8–12 weeks old) were purchased from SLC (Hamamatsu, Japan). All mice were treated humanely according to the Guide for the Care and Use of Laboratory Animals, National Institutes of Health. All mice were housed in a temperature-controlled environment (24 ± 1 °C) with a humidity level of 55 ± 5% and alternating 12 h light/dark cycles. They had free access to water and standard rodent food.

### 2.2. Tissue Preparation

Pregnant ICR mice (8–12 weeks old) were injected subcutaneously with romiplostim (Nplate ^®^, Amgen; 1, 5, 10, 30, 100 µg/kg) on gestational days (GD) 1, 8, and 15 (GD1 was the day after vaginal plugs were detected). Blood samples from pregnant mice were collected on GD18.

Blood from pregnant mice was collected (6 µL) using a micropipette with a 25-gauge needle disrupting the lateral tail vein and coated with ethylenediaminetetraacetic acid (EDTA) on the tip. The blood was collected in microtubes containing EDTA with phosphate-buffered saline (PBS 30 µL). Blood cell counts were performed within 1 h using a fully automated animal hematology analyzer MEK-6450 (Nihon Kohden, Tokyo, Japan). On GD18, the pregnant mice were anesthetized with isoflurane (inhaled 1.5–3%) and sacrificed by cervical dislocation. Their femoral bone, fetal blood, fetal liver, and placenta were then collected. The rate of resorption, the number of pups per litter, fetal weight at birth, and placental weight were also recorded. Fetal blood was collected using a micropipette by disrupting the anterior facial vein with a 25-gauge needle (10 µL) and was collected in microtubes containing EDTA in phosphate-buffered saline (30 µL). Blood cell counts were then counted. The placentas were either frozen and stored at −80 °C (for RNA analysis) or fixed in formalin for 24 h and stored in 70% ethanol (for morphology) until embedded in paraffin using standard procedures.

### 2.3. Histology

Tissues from the placenta and fetal liver were fixed in formalin, embedded in paraffin and then sectioned at 4 µm. For morphological analysis, the sections were stained with hematoxylin and eosin (H&E). The femoral bone from the pregnant mice was decalcified with Decalcifying Solin A (Fujifilm, Osaka, Japan) for 12 h, then embedded in paraffin and stained with H&E.

### 2.4. Counting of Megakaryocytes

Megakaryocytes (MK) are 50 to 100 µm in diameter, 10 to 15 times larger than red blood cells, and their large, migrating nucleus makes visual inspection and counting by microscopy easy. On each slide, the MK frequency was calculated as the mean of the number of MKs in each field (the total number of MK scores divided by the total number of fields evaluated).

### 2.5. Measurement of the Vessel Wall of the Spiral Artery

The inner and outer diameters of the thinnest part of the spiral artery in the H&E-stained transverse image of the basal layer of the placenta were measured.

The thickness of the spiral arterial wall was calculated as: (outer diameter-inner diameter)/2.

### 2.6. RNA Extraction, cDNA Synthesis, and Quantitative PCR (qPCR)

RNA was extracted from the frozen placenta samples using the RNeasy Plus Mini Kit (Qiagen, Hilden, Germany) according to the manufacturer’s protocol. qPCR was performed using the comparative threshold cycling method to determine glyceraldehyde-3-phosphate dehydrogenase. Primers and probes for vascular endothelial growth factor (VEGF)-related genes (soluble FMS-like tyrosine kinase 1 (sFLT1): ABI Assay ID Mm00438980_m1, placental growth factor (PlGF): Mm00435613_m1, VEGF-A: Mm00437306_m1, and VEGF-B: Mm00442102_m1) were obtained from Thermo Fisher Scientific Inc (Thermo Fisher Scientific, Waltham, MA, USA). The relative quantification was normalized to the glyceraldehyde 3-phosphate dehydrogenase gene (GAPDH: Mm99999915_g1) expression.

### 2.7. Statistical Analysis

All data of medians and range were presented. Comparisons among each group were made by the Mann–Whitney U test or the Student’s *t*-test. Differences were considered statistically significant at *p* < 0.05, and the data were analyzed via SPSS 21 software (SPSS Inc., Chicago, IL, USA).

## 3. Results

**Figure 1:**
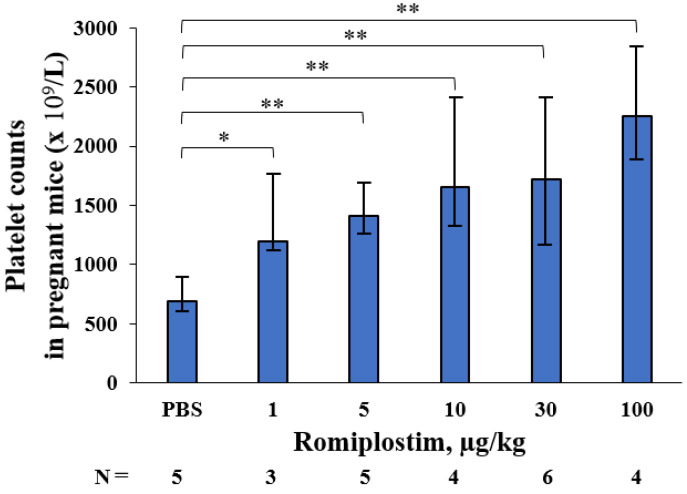
Platelet counts in pregnant mice treated with romiplostim on GD18. Romiplostim significantly increased platelet counts in pregnant mice dose-dependently. Pregnant mice were administered romiplostim (1, 5, 10, 30, and 100 µg/kg) subcutaneously on GD1, 8, and 15, and platelet counts were measured on GD18. Bars represent the median. Error bars represent range. * *p* < 0.05, ** *p* < 0.01.

**Figure 2:**
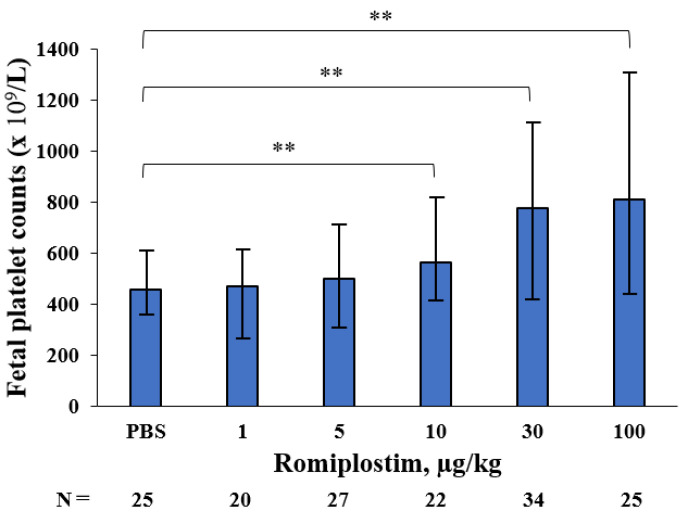
Fetal platelet counts of pregnant mice treated with romiplostim. Fetal platelet counts of pregnant mice treated with romiplostim (10, 30, and 100 µg/kg) significantly increased dose-dependently compared to the control group. Bars represent the median. Error bars represent range. ** *p* < 0.01.

**Figure 3:**
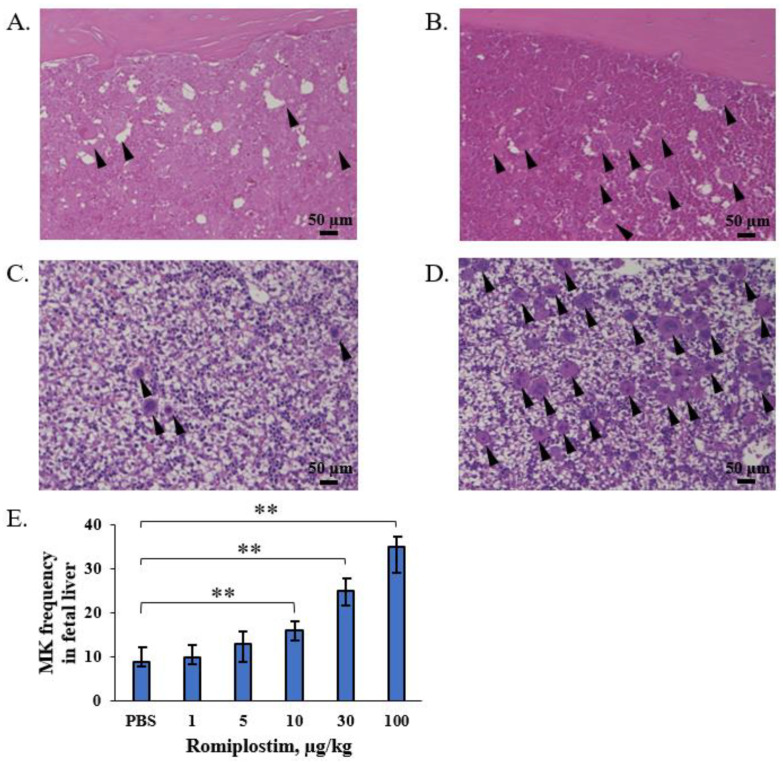
MK in maternal femoral bone marrow and fetal liver. Femoral bone marrow histology (H&E) in the control pregnant mice (**A**) and pregnant mice treated with 100 µg/kg romiplostim (**B**) on GD18. Liver histology (H&E) of fetuses of control pregnant mice (**C**) and of pregnant mice treated with 100 µg/kg romiplostim (**D**). The black arrowheads mark megakaryocytes. Scale bars 50 µm, 20 magnification. (**E**) MK frequency (the total number of MKs divided by the total number of scorable fields) under 20 magnification is shown for PBS, 1, 5, 10, 30, and 100 µg/kg romiplostim in fetal liver section triplicate experiments (N = 5). Bars represent the median. Error bars represent range. ** *p* < 0.01.

**Figure 4:**
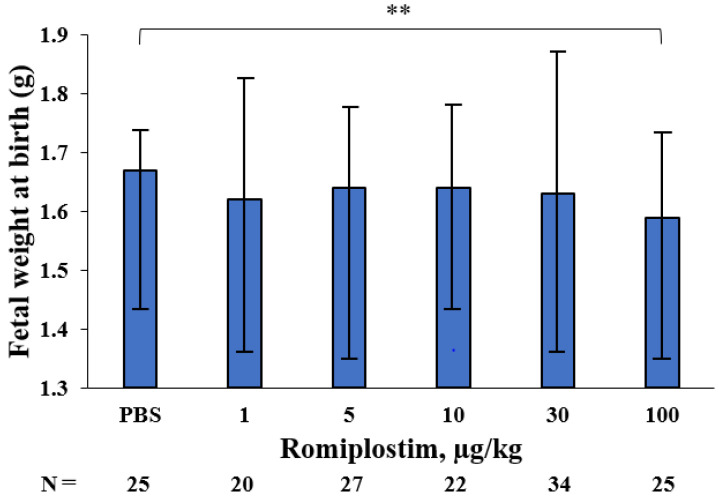
Fetal weight at birth of pregnant mice treated with romiplostim. The administration of 100 µg/kg of romiplostim to pregnant mice significantly reduced fetal weight at birth, compared to the control group. Bars represent the median. Error bars represent range. ** *p* < 0.01.

**Figure 5:**
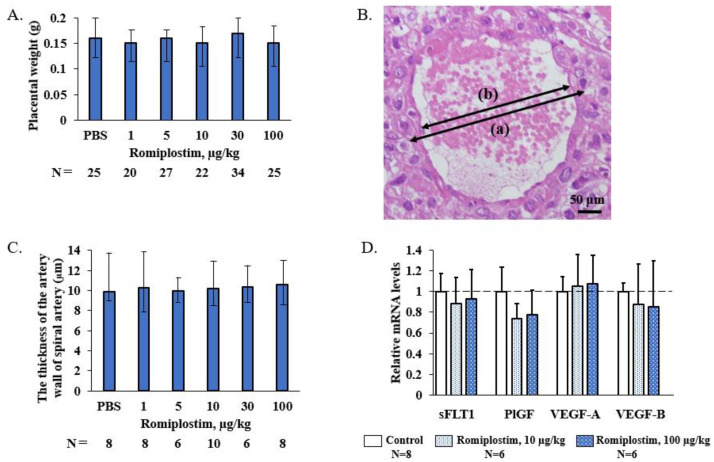
Placental weight, the spiral arteries of basal decidua, and RNA expression of VEGF signaling in placentas on GD18. (**A**) There was no significant difference in placental weight in the romiplostim treated group compared to the control group. Bars represent the median. Error bars represent range. (**B**) The thickness of the arterial wall of the spiral artery was calculated as: ((a) outer diameter-(b) inner diameter)/2. Scale bars 50 µm, 20 magnification. (**C**) There was no significant difference in the thickness of the arterial wall of the spiral artery in the romiplostim treated group compared to the control group. Bars represent the median. Error bars represent range. (**D**) There was no significant difference in the mRNA expression levels of sFLT1, PlGF, VEGF-A, and VEGF-B by quantitative PCR in the placenta of pregnant mice treated with romiplostim compared to the control group. Bars represent the mean. Error bars represent standard deviation.

When the pregnant mice were treated with romiplostim (1, 5, 10, 30, and 100 µg/kg) on GD1, 8, and 15, their median platelet counts (×10^9^/L) increased to 1194, 1410, 1659, 1716 and 2256 on GD18, respectively, compared to 692 in the control group (Figure 1). The fetuses of the pregnant mice treated with 1 and 5 µg/kg doses of romiplostim did not show a significant increase in median platelet counts (×10^9^/L), but at doses of 10 µg/kg and higher, they showed a significant dose-dependent increase in median platelet counts: 562, 776, and 812, respectively, compared to 456 in the control group (Figure 2). In the histological images (H&E) of femoral bone marrow of the pregnant mice treated with 100 µg/kg of romiplostim on GD18, a prominent increase in MK counts could be observed, compared to the control group (Figure 3A,B). The increased platelet counts in the fetuses also corresponded to the increased MK counts in the fetal liver (Figure 3C,D). MK frequency in the fetal liver was significantly increased at doses above 10 µg/kg compared to the control group, as were the fetal platelet counts (Figure 3E). The change in MK frequency in the fetal liver reflected an increase in fetal platelet counts. When pregnant mice were treated with 100 µg/kg of romiplostim, the median fetal weight at birth was significantly decreased compared to the control group (1.67 vs. 1.59 g, respectively; *p* < 0.01; Figure 4), although no significant change was recognized in placental weight (Figure 5A). We also evaluated the effects of romiplostim on pregnant mice with regard to the rate of resorption and the number of pups per litter, and we found no significant differences compared to the control group (Table 1). We evaluated the thickness of the vessel wall of the spiral artery in the basal layer of the placenta as well (Figure 5B), and no significant changes were observed in either group (Figure 5C). The expression of sFLT1 and VEGF mRNAs in the placenta on GD18 was analyzed by qPCR. The mRNA level of sFLT-1 exhibited no clear up or down regulation among three groups (control, 10 and 100 µg/kg). Growth factors such as PlGF, VEGF-A, and VEGF-B also did not show significant changes (Figure 5D).

## 4. Discussion

When pregnant mice were treated with romiplostim (1, 5, 10, 30, and 100 µg/kg) on GD1, 8, and 15, maternal platelet counts on GD18 were increased at all doses. There was also an increase in megakaryocyte counts in femoral bone marrow. In fetuses, a dose-dependent increase in platelet count was observed at doses of 10 µg/kg and higher, and an increase in the megakaryocyte count in the liver was observed at doses similar to those which affected the platelet count. The extremely high dose of romiplostim (100 µg/kg) significantly decreased the fetal weight at birth but did not affect the miscarriage rate. In the placentas of pregnant mice treated with romiplostim, there was no difference in weight compared to the controls, and there was no thickening or thinning of the spiral artery wall in the placental basal layer, thus indicating that remodeling failure of the spiral artery did not occur. This may be due to the fact that there was no signal change in the mRNA of placental vascular growth factors sFLT1, PlGF, VEGF-A, and VEGF-B.

In the administration of romiplostim to non-pregnant mice, it has been reported that a single subcutaneous dose of romiplostim (AMP4, Amgen, 0.1, 1, and 10 µg/kg) significantly increased platelet counts at 72 h post-dose at 1 and 10 µg/kg [11]. Our study is the first paper to confirm a maternal platelet increase after the administration of romiplostim to mice throughout pregnancy and at a wider range of concentrations. Additionally, romiplostim at doses of 10 µg/kg or higher was found to increase platelet and megakaryocyte counts in the fetal liver. The hematopoietic site of fetal mice switches from the yolk sac to the liver and bone marrow with the number of gestational weeks.

As the hematopoietic stem cells (HSCs) settle from the yolk sac into the liver bud at around embryonic day 11, the number of fetal liver HSCs increases dramatically until embryonic day 15.5. Fetal liver HSCs then disappear 2–4 days after delivery, and it is believed that the hematopoietic site shifts to the bone marrow [12]. We evaluated the megakaryocyte count in the fetal liver on GD18, when hematopoiesis is active in the liver, and found an increase in the megakaryocyte count, thus indicating that romiplostim acts on the fetal liver to increase the platelet count.

A significant decrease in fetal weight at birth was observed only at an extremely high dose of 100 µg/kg of romiplostim. There have been no reports thus far that have shown a decrease in fetal weight at birth after the administration of romiplostim to animals. We sought to determine if the decrease in fetal weight at birth was due to inadequate remodeling of the spiral arteries, which causes fetal growth restriction (FGR) and miscarriage of fetuses. Inadequate remodeling of spiral arteries is caused by shallow trophoblast invasion and insufficient vasodilation of spiral arteries while maintaining their autoregulatory function, thus resulting in inadequate blood volume to perfuse the placenta and fetuses and therefore leading to FGR and miscarriage [13]. In our study, the thickness of the spiral artery wall was unchanged at all doses, compared to the controls. Factors that affect spiral artery remodeling, subsequent placentation and fetal development, such as sFLT1, PlGF, VEGF-A, and VEGF-B, were examined, and no signal changes in mRNA were observed. We also administered romiplostim throughout pregnancy, starting before GD8–10, when remodeling occurred in the mouse placenta [14]. This suggests that the reduced fetal weight at birth was not due to inadequate remodeling of the spiral arteries, which may have resulted in no difference in miscarriage rates. The decrease in fetal weight at birth was seen only at an extremely high doses of 100 µg/kg, and doses of 1–10 µg/kg used in cases of ITP are considered to have little effect on fetal weight at birth. On the other hand, Suzuki et al. reported an infant born with a low birth weight in a case of human pregnancy treated with eltrombopag [15]. However, we did not investigate the effect of eltrombopag in this study due to its limited specificity and activity on humans and chimpanzees [16].

ITP is an autoimmune disease that results in platelet depletion due to increased platelet destruction and impaired platelet production caused by immune mechanisms such as autoantibodies against platelets. Approximately 15% of patients with severe, multi-resistant ITP die from complications due to severe bleeding [17,18], and the mortality rate is 1.3 to 2.2 times higher than that of the general population [19]. Refractory ITP is a serious, life-threatening disease, but since the approval of TPO-RAs, it has been reported that more than 80% of patients who are refractory to both steroids and IVIg responded to romiplostim [20,21], indicating that romiplostim has been shown to be effective in the treatment of refractory ITP. The first-line therapy for pregnant women with ITP is limited to IVIg and corticosteroids for safety reasons, including fetal teratogenicity [22]. However, there are cases of refractory ITP that do not respond to these therapies. Thus, it is very important to confirm the safety of treatment with TPO-RAs in human pregnant women with ITP. To the best of our knowledge, nine cases of romiplostim use in human pregnant women were found [23,24,25,26,27,28,29]. Among them, there were two reports of romiplostim administration in cases of systemic lupus erythematosus and mixed connective tissue disease, in addition to ITP [27]. None of the doses of romiplostim were less than 6 µg/kg. Maternal platelet counts increased after romiplostim administration in eight of nine patients, and of the eight patients who responded to treatment, one had severe bleeding during delivery, which was attributed to gestational hypertension with serious complications for the mother and infant [28]. There were two reports of patients diagnosed with ITP before pregnancy who were refractory to steroids, IVIg, rituximab, other immunosuppressive drugs, and splenectomy, but responded well to romiplostim and used it throughout pregnancy [23]. The other seven patients had started romiplostim treatment after the late second trimester at the onset of symptoms such as epistaxis and gingival bleeding or preparation for delivery. In three cases, the fetal weight at birth was not described. However, in the other cases, FGR was not observed and perinatal outcomes were good for both mother and newborn. Although case reports on the administration of romiplostim on human pregnant women with refractory ITP are still few, and further findings are needed, no apparent adverse effects have been observed so far.

In the present study, platelet and megakaryocyte counts of the fetuses were increased, thus indicating that romiplostim acts on the fetal liver to increase the platelet count, which is transferred to the fetus through the placenta. This study also suggests that extreme doses may decrease fetal weight at birth. The cause of the decrease in fetal weight at birth and the long-term prognosis of fetuses are not clear, and the major limitation of this paper is that it was not administered to an ITP model mouse. However, this is the first paper to evaluate maternal platelet and megakaryocyte counts, fetal weight at birth, placental morphology, and miscarriage rates after the administration of romiplostim to pregnant mice at human doses and at higher doses throughout pregnancy.

## 5. Conclusions

Although the results of the present study are encouraging, until there are more conclusive data, the use of romiplostim should be evaluated individually in severe, life-threatening cases, and all relevant ethical aspects should be considered.

## Figures and Tables

**Table 1:** The rate of resorption and the number of pups per litter when romiplostim was administered to pregnant mice.

Romiplostim	Rate of Resorption (%) Median (Range)	Pups/Litter Median (Range)
PBS	0 (0–16.7)	11 (9–16)
1 µg/kg	0 (0–23.1)	16 (10–16)
5 µg/kg	7.1 (0–14.3)	13 (12–14)
10 µg/kg	7.7 (7.1–20.0)	12 (12–13)
30 µg/kg	6.9 (0–9.1)	13 (10–15)
100 µg/kg	8.7 (7.1–20.0)	12 (10–16)

Administration of romiplostim to pregnant mice did not significantly affect the rate of resorption or the number of pups per litter (Mann–Whitney U test, *p* < 0.05).

## Data Availability

The study did not report any data.
